# Giant Adrenal Cyst: A Case Report

**DOI:** 10.7759/cureus.37086

**Published:** 2023-04-03

**Authors:** Nengfeng Yu, Jiaqi Du, Gangfu Zheng, Yichun Zheng

**Affiliations:** 1 Department of Urology, The Fourth Affiliated Hospital, Zhejiang University School of Medicine, Yiwu, CHN

**Keywords:** the diagnosis of adrenal cysts, the surgical indication of adrenal cysts, the management of adrenal cysts, angiomatous adrenal endothelial cyst, adrenal cysts

## Abstract

Giant adrenal cysts are rare lesions, most often discovered incidentally. In this case report, a patient presenting with nonspecific abdominal distension is described. Imaging studies revealed a vast cystic mass closely attached to the left adrenal gland. Neither routine laboratory tests nor endocrine function tests revealed abnormalities. By performing open surgery, the cystic mass was completely removed. According to the pathological results, the wall of the cystic mass has an endothelial structure and some vascular components. Comprehensive analysis indicated that this case was an angiomatous adrenal endothelial cyst which was an extremely uncommon form of an adrenal cyst. Over a one-year follow-up, no evidence of recurrence was observed in the patient postoperatively. Through this case, we wish to raise awareness of this disease.

## Introduction

Adrenal cysts are relatively uncommon and asymptomatic, with a reported incidence rate of 0.06-0.18% [1]. An increasing number of adrenal cysts are discovered incidentally, thanks to improved radiologic imaging techniques. Size and symptoms of adrenal cysts, as well as whether they are functioning or potentially malignant, determine how they are managed. An accurate diagnosis needs the help of pathological examination [1,2].

## Case presentation

A 38-year-old female was admitted to the hospital owing to abdominal distension for about one year without special abdominal pain or other discomforts. On physical examination, a large mass was found on the left side of the abdomen, accompanied by firm texture, smooth surface, and well-defined borders. There was no tenderness or knock pain in the whole abdomen. There were no abnormalities in the routine laboratory tests or the endocrine function tests.

**Figure 1:**
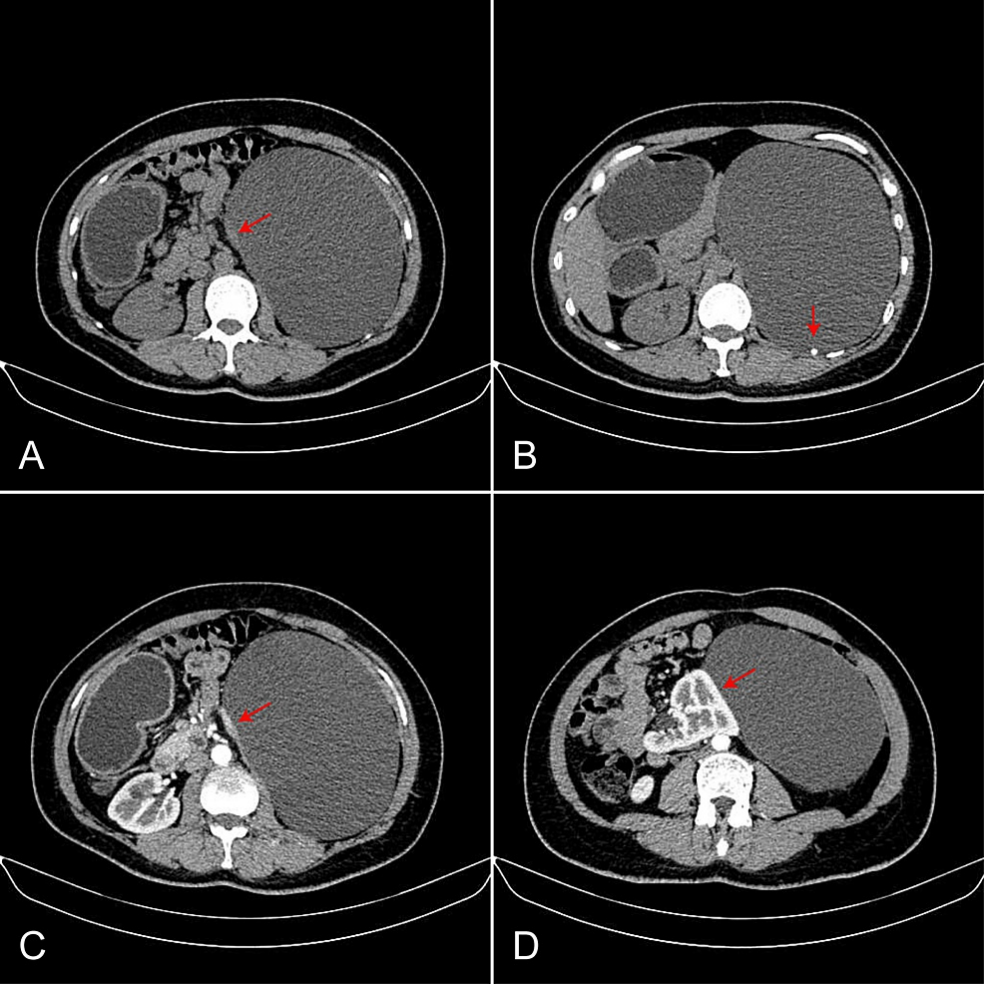
The imaging features of the cystic mass on computerized tomography scan and enhanced scan. (A) The imaging shows a giant cystic lesion with localized cystic wall thickening (arrows). (B) The imaging shows a nodular calcification in the cyst wall (arrows). (C) The imaging shows obvious enhancement of the focal thickened cystic wall, suggesting the left adrenal gland (arrows). (D) The imaging shows the relationship between the cystic lesion, the deformed kidney (arrows), and surrounding organs.

On abdominal computed tomography (CT) imaging, a 31×17×16cm giant unilocular cystic lesion with a clear boundary was seen in the left retroperitoneal space (Figure 1A). The lesion had nodular calcification in the cyst wall (Figure 1B). After the injection of the contrast medium, the cystic areas were non-enhanced, but the focal thickened cyst wall showed obvious enhancement (Figure 1C). The enhancement degree of the cyst wall was close to normal adrenal gland tissue, which suggested the lesion probably originated from the left adrenal gland. The compressive effect of the mass resulted in the displacement of the left kidney to the right (Figure 1D).

**Figure 2:**
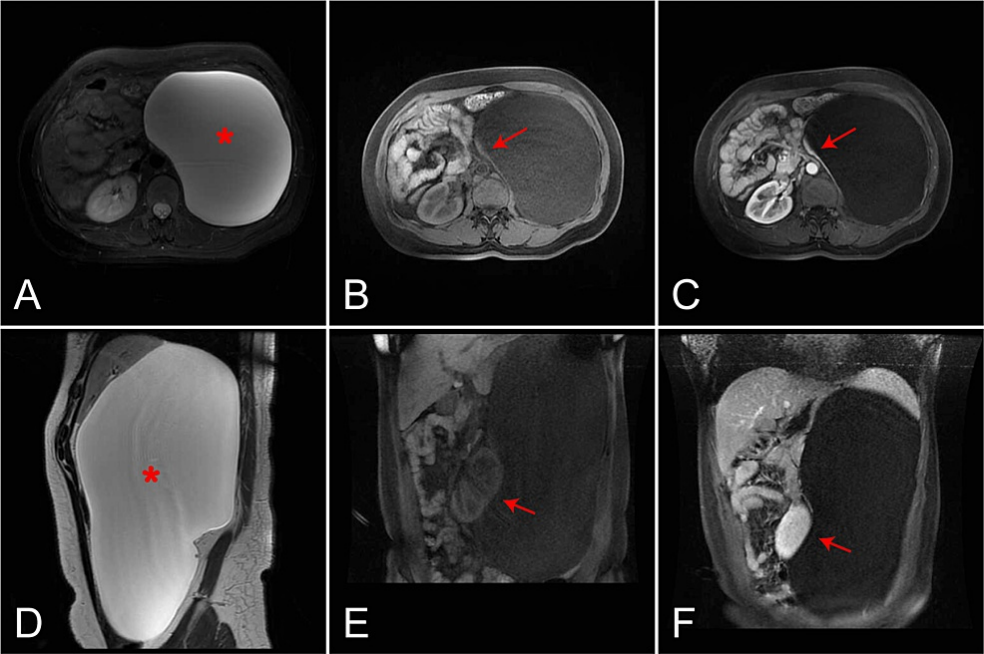
The imaging features of this cystic mass on magnetic resonance imaging. Axial (A) and Sagittal (D) T2-weighted MR images of the cystic mass demonstrate a predominantly high signal-intensity lesion (asterisk). Axial T1- (B) and enhanced T1-weighted (C) images show rim enhancement of the lesion, suggesting the left adrenal gland (arrows). Coronal T1- (E) and enhanced T1-weighted (F) images show the displaced kidney (arrows).

On MRI, the cystic mass displayed high intensity in T2-weighted images (Figures 2A, 2D) and low intensity in T1-weighted images (Figures 2B, 2E), indicating the presence of serous fluid inside. Similarly, sagittal T2-weighted and coronal T1-weighted images demonstrated a prominent mass with significant compression of adjacent structures (Figures 2D, 2E). Postcontrast imaging with gadolinium showed rim enhancement of the focal lesion but absent central enhancement (Figures 2B, 2C, 2E, 2F).

Due to the huge volume and the risk of malignancy, we adopt an open excision. Anesthesia was administered to the patient, and he was placed supine on the operating table. Following an abdominal incision, intraoperative examination revealed that the cystic mass was closely adherent to the left kidney and left adrenal gland, and there was significant compression of the left kidney, left adrenal gland, pancreas, spleen, jejunum, and ileum. Dissociation was made from below to above against the cystic wall of the mass. And then, the cystic mass was completely excised. There were no complications during the operation during the perioperative period.

**Figure 3:**
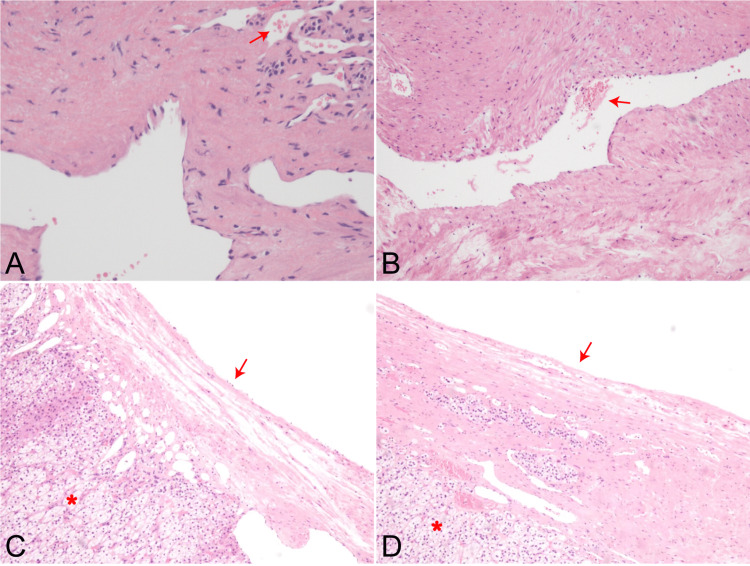
Hematoxylin-eosin staining results of the cystic mass. (A) Small blood vessel components are shown under a 100X microscope (arrows). (B) The vascular component of the other part is shown under a 40X microscope (arrows). (C) Adrenal acinar cells (asterisk) and the capsule wall with scattered endothelial cell lining (arrows) are shown under a 40X microscope. (D) Adrenal acinar cells (asterisk) and the smooth capsule wall without endothelial cell lining (arrows) are shown under a 40X microscope.

Based on cytological analyses of the cyst fluid, no malignant component was found. It appears that this cystic mass has some vascular components according to hematoxylin-eosin staining (Figures 3A, 3B), and the part cyst wall consisted of a single flattened cell lining supposed to be derived from endothelium or mesothelium (Figures 3C, 3D). According to hematoxylin-eosin staining, the capsule wall closely adhered to the adrenal acinar tissue (Figures 3C, 3D). This indicated that the cyst might originate from the adrenal gland. Immunohistochemical results showed the immunopositivity of ERG (vascular endothelium) and the negativity of D2-40. After a comprehensive analysis, the final diagnosis was considered as an angiomatous type of adrenal endothelial cyst. During the one-year follow-up period, there were no residual abdominal symptoms nor radiographic evidence of recurrence observed.

## Discussion

A benign adrenal cyst is a rare lesion of the adrenal gland that makes up less than 1-2% of all adrenal incidentalomas [3,4], the overall prevalence is about 0.06-0.18% [1]. Most benign adrenal cysts are discovered incidentally with mild symptoms [5]. Because of the large potential retroperitoneal space, adrenal cysts are clinically asymptomatic in their early stages like other primary retroperitoneal tumors [6]. The onset of clinical symptoms often indicates that the mass has grown in size or that complications have developed, such as this case. In addition to non-specific abdominal distension, abdominal pain is also one of the most common symptoms. When the abdominal viscera and blood vessels are compressed, there may be clinical manifestations such as nausea, vomiting, discomfort after eating, constipation, and lower extremity edema [6]. A unique danger of adrenal cysts is that impacting the patient's abdomen may cause acute abdomen or even shock if the cyst ruptures [7]. In our case, the cyst had enlarged and produced a “mass effect”, resulting in nonspecific abdominal distension. However, due to the large space of the retroperitoneum, its symptoms were still mild. In addition to clinical symptom evaluation, an endocrinological examination is also an important part of disease management. It is necessary to ascertain whether the cyst has abnormal endocrine activity. Benign adrenal cysts are generally non-functional lesions [1,2]. It is important to investigate all adrenal lesions for endocrine activity and malignancy features due to the possibility of unknown endocrine disorders or malignancies [2,5]. Abnormal endocrine activity often indicates the need for surgical treatment [2].

After accurate clinical and functional evaluation, imaging examinations are performed to evaluate the size, morphological features, and relations with surrounding structures [2]. Adrenal cysts are mainly round or oval lesions with a clear boundary and homogenous appearance [1]. Their median size is about 57.5-58 mm [1,2]. Adrenal cysts with a diameter of over 200mm are very rare. In contrast to most benign adrenal cysts, only a few patients showed a slight intracystic enhancement with contrast. A study showed that a median unenhanced attenuation measured in 76 patients was 19 HU (range, 0 to 83 HU) [1]. Magnetic resonance imaging (MRI) is an appropriate tool for determining the origin and morphology of tumors [8]. When it comes to giant adrenal cysts, magnetic resonance imaging (MRI) is more effective than CT scans. On MRI, the adrenal cysts are usually found to be high intensity in T2-weighted images with a thin wall. It indicates that the interior of the cyst is filled with serous fluid [9]. These imaging features are similar to the imaging findings of our case.

Although ultrasonographic scans, computed tomographic scans, and magnetic resonance images could demonstrate the cystic nature and liquid content of these cysts, their histologic type cannot be identified. Adrenal cysts have traditionally been classified into endothelial, pseudocyst, epithelial, and parasitic cysts based on histopathology [10]. In most cases, adrenal cysts are endothelial cysts, which can be classified as angiomatous or lymphatic cysts. Angiomatous endothelial cysts are extremely rare [9]. CD31, CD34, Fli-1, and Factor VIII are positive markers of endothelial cells [11]. It is possible to distinguish the lymphatic type by the immunopositivity of D2-40 and the negativity of CALRTININ, as well as WT1 [9,12]. The final pathological diagnosis of our case depended on the immunopositivity of ERG (vascular endothelium) and the negativity of D2-40. Histology and immunohistochemistry are helpful for the accurate diagnosis of adrenal cysts. 

As one kind of benign lesion, adrenal cysts can choose expectant treatment in small size with no clinical symptoms. Functional cysts and malignant or potentially malignant cysts are usually surgically treated. Cysts over 5 cm and symptomatic cysts smaller than 5 cm are also indications for surgery [2,13]. Although it is more recommended to enucleate the cyst laparoscopically while preserving the adrenal gland. When there is a possibility of malignancy or complex adrenal cyst, the surgical procedure should be chosen after a thorough evaluation and a comprehensive analysis [8,9] With a huge cyst like the present case, we opted for open surgery for complete resection. Studies have shown that 7% of cysts are malignant and any rupture exposes patients to the risk of peritoneal dissemination [2,14]. This is also an important factor affecting the deterioration of prognosis [14]. Thus, the basic principle is complete excision without destroying the integrity of the mass as much as possible. Although the prognosis for adrenal cysts is excellent, there still exists a risk of recurrence in some cases, even after complete resection [15,16]. Therefore, long-term follow-up is recommended.

## Conclusions

Adrenal cysts are exceptionally rare, especially giant angiomatous type of adrenal endothelial cysts. It is challenging to differentiate an adrenal cyst from other adrenal cystic masses. As the first step, an endocrine assessment should be performed for evaluation. A CT scan and MRI are also essential for identifying adrenal cystic masses. The surgical treatment is indicated for functional cysts, malignant cysts, potentially malignant cysts, symptomatic cysts, as well as asymptomatic cysts larger than 5 cm.
